# Photosystem I light-harvesting proteins regulate photosynthetic electron transfer and hydrogen production

**DOI:** 10.1093/plphys/kiac055

**Published:** 2022-02-14

**Authors:** Thi Thu Hoai Ho, Chris Schwier, Tamar Elman, Vera Fleuter, Karen Zinzius, Martin Scholz, Iftach Yacoby, Felix Buchert, Michael Hippler

**Affiliations:** Institute of Plant Biology and Biotechnology, University of Münster, Münster 48143, Germany; Faculty of Fisheries, University of Agriculture and Forestry, Hue University, Hue 530000, Vietnam; Institute of Plant Biology and Biotechnology, University of Münster, Münster 48143, Germany; School of Plant Sciences and Food Security, The George S. Wise Faculty of Life Sciences, Tel Aviv University, Tel Aviv 69978, Israel; Institute of Plant Biology and Biotechnology, University of Münster, Münster 48143, Germany; Institute of Plant Biology and Biotechnology, University of Münster, Münster 48143, Germany; Institute of Plant Biology and Biotechnology, University of Münster, Münster 48143, Germany; School of Plant Sciences and Food Security, The George S. Wise Faculty of Life Sciences, Tel Aviv University, Tel Aviv 69978, Israel; Institute of Plant Biology and Biotechnology, University of Münster, Münster 48143, Germany; Institute of Plant Biology and Biotechnology, University of Münster, Münster 48143, Germany; Institute of Plant Science and Resources, Okayama University, Kurashiki, Japan

## Abstract

Linear electron flow (LEF) and cyclic electron flow (CEF) compete for light-driven electrons transferred from the acceptor side of photosystem I (PSI). Under anoxic conditions, such highly reducing electrons also could be used for hydrogen (H_2_) production via electron transfer between ferredoxin and hydrogenase in the green alga *Chlamydomonas reinhardtii*. Partitioning between LEF and CEF is regulated through PROTON-GRADIENT REGULATION5 (PGR5). There is evidence that partitioning of electrons also could be mediated via PSI remodeling processes. This plasticity is linked to the dynamics of PSI-associated light-harvesting proteins (LHCAs) LHCA2 and LHCA9. These two unique light-harvesting proteins are distinct from all other LHCAs because they are loosely bound at the PSAL pole. Here, we investigated photosynthetic electron transfer and H_2_ production in single, double, and triple mutants deficient in PGR5, LHCA2, and LHCA9. Our data indicate that *lhca2* and *lhca9* mutants are efficient in photosynthetic electron transfer, that LHCA2 impacts the *pgr5* phenotype, and that *pgr5/lhca2* is a potent H_2_ photo-producer. In addition, *pgr5*/*lhca2* and *pgr5*/*lhca9* mutants displayed substantially different H_2_ photo-production kinetics. This indicates that the absence of LHCA2 or LHCA9 impacts H_2_ photo-production independently, despite both being attached at the PSAL pole, pointing to distinct regulatory capacities.

## Teaser

Alteration of the light-harvesting composition of photosystem I impacts photosynthetic electron transfer and hydrogen production.

## Introduction

Oxygenic photosynthesis drives the conversion of solar energy into chemical energy and building material. The accompanied production of oxygen and the assimilation of carbon dioxide largely determines the composition of Earth’s atmosphere and surface (Nelson and Yocum, 2006). This energy conversion process is catalyzed by four multi-subunit membrane protein complexes, which are embedded into thylakoid membranes of cyanobacteria and chloroplasts of eukaryotic photosynthetic organism: photosystem I (PSI), photosystem II (PSII), the cytochrome (Cyt) *b_6_f* complex, and ATPase (Nelson and Yocum, 2006). Among these complexes, PSI possesses the most negative redox potential in nature. PSI is a light-driven plastocyanin: ferredoxin (FDX) oxidoreductase. Electron transfer from FDX to the FDX NADPH oxidoreductase (FNR) results in the formation of NADPH, that is the final product of linear electron flow (LEF). Alternatively, FDX may be involved in re-directing electron transfer into Cyt *b_6_f* complex and/or NADPH oxidases (Joliot and Joliot, 2006; Yamamoto et al., 2011). The resulting cyclic electron flow (CEF) will provide an ATP poise and protect the PSI acceptor side from over-reduction. CEF around PSI was first recognized by Arnon (1959). However, its mechanistic mode of action is not yet clear. CEF may involve the direct reduction of plastoquinone (PQ) via a NAD(P)H dehydrogenase (NDH)-dependent electron transfer. In *Chlamydomonas reinhardtii* this is catalyzed by a type II NAD(P) dehydrogenase (NDA2) (Jans et al., 2008; Desplats et al., 2009). Alternatively, in chloroplasts, a PROTON-GRADIENT REGULATION5 (PGR5)-related pathway (Shikanai, 2007; Alric, 2010; Peltier et al., 2010) may be involved in PQ reduction or in a direct reduction of a quinone bound to the Q_i_ site of Cyt *b*_6_*f* by combined electron transfer from its proximal heme c_i_ and an FNR bound to the complex (Joliot et al., 2004; Buchert, 2020). Remodeling processes of PSI, including proteins of the PSI-associated light-harvesting complex (LHCI) may also be involved in partitioning of LEF and CEF. Indeed, there is evidence of a protein supercomplex composed of PSI-LHCI, LHCII, the Cyt *b_6_f* complex, and FNR isolated from *C. reinhardtii* (Iwai et al., 2010). Recently, additional structural data provided evidence for a PSI-Cyt *b_6_f* supercomplex (Steinbeck et al., 2018). In this work, formation of a PSI-Cyt *b_6_f* supercomplex was linked to the remodeling of PSI-associated light-harvesting proteins (LHCAs), particularly via unbinding of LHCA2 and LHCA9 from PSI. LHCA2 and LHCA9 occupy LHC binding sites at the PSAL pole, which have been identified in PSI structures from red and green algae (Ozawa et al., 2018; Pi et al., 2018; AntoshviLi et al., 2019; Kubota-Kawai et al., 2019; Qin et al., 2019; Su et al., 2019; Suga et al., 2019). While PSI from vascular plants contains 4 LHCs (Mazor et al., 2015, 2017; Qin et al., 2015; Pi et al., 2018), the PSI of green algae may contain up to 10 LHCs; 2 at the PSAL pole and up to 8 arranged in two crescents at the PSAF pole (pointing toward the lumen) (Ozawa et al., 2018; Kubota-Kawai et al., 2019; Qin et al., 2019; Su et al., 2019; Suga et al., 2019). Notably, a resembling organization was also reported in vascular plants, where an additional LHCA1–A4 dimer was found to be bound on the PSAL side of *Arabidopsis thaliana* (Crepin et al., 2020), suggesting that this mode of organization is also shared with vascular plants. In a recent cryogenic electron microscopy study, Naschberger et al. (2021) identified a PSI dimer from *C. reinhardtii* where two copies of LHCA9 tethered two monomeric PSI in a head-to-head fashion, forming a large oligomeric protein complex. In this structure, LHCA2 and PSAH were absent. This further underpins the potential role of LHCA2 and LHCA9, both binding at the PSAL pole, in functional remodeling of PSI. LHCA2 and LHCA9 are also involved in the formation of a PSI–LHCI–LHCII complex (Pan et al., 2021). A schematic view on PSI remodeling processes is provided (please see below).

Functional remodeling of PSI may also be involved in hydrogen photo-production in *C. reinhardtii*, where FDX1 donates photosynthetic electrons to a chloroplast localized hydrogenase (HYDA) that produces H_2_ (Happe et al., 2002). H_2_ photo-production in the green alga is observed under sulfur deprivation (Nagy et al., 2018; Melis et al., 2000), which leads to substantial degradation of the photosynthetic apparatus, including PSII. This induces anoxia and expression of the hydrogenase enzyme HYDA, initiating H_2_ photo-production for several days. In a different strategy, a light pulse protocol was developed, which allowed H_2_ production at high light-to-H_2_ conversion efficiency supporting H_2_ production during the light cycles (Kosourov et al., 2018). To minimize the inhibitory effect of O_2_ on HYDA activity and thereby sustain high H_2_ photo-production, low O_2_ concentration level can be accomplished by employing an iron-salt based O_2_ absorbent in the headspace (Nagy et al., 2018) or adding a mixture of ascorbate and copper to the alga culture (Khosravitabar and Hippler, 2019). Different *C. reinhardtii* mutant strains have been identified which produce significantly more H_2_ as compared to respective wild-type (WT) strains (Toth and Yacoby, 2019). Notably, most prominent H_2_ photo-production in *C. reinhardtii* was observed in a PGR5-deficient mutant, both under sulfur deprivation as well as under anoxia (Steinbeck et al., 2015; Nagy et al., 2021), indicating a strong link between the capacity of H_2_ production and regulation of photosynthetic electron transfer.

In this article, we provide evidence that deletion of LHCA2 impacts PGR5-dependent regulation of photosynthetic electron transfer and boosts light-driven H_2_ production in a *pgr5* KO mutant. These results are discussed in the framework of PSI remodeling processes, where LHCA2 and LHCA9 appear to play regulatory functions.

## Results

### Generation and proteomic analyses of *lhca2*, *lhca9*, and *pgr5* single, double, and triple mutant strains

**Figure 1:**
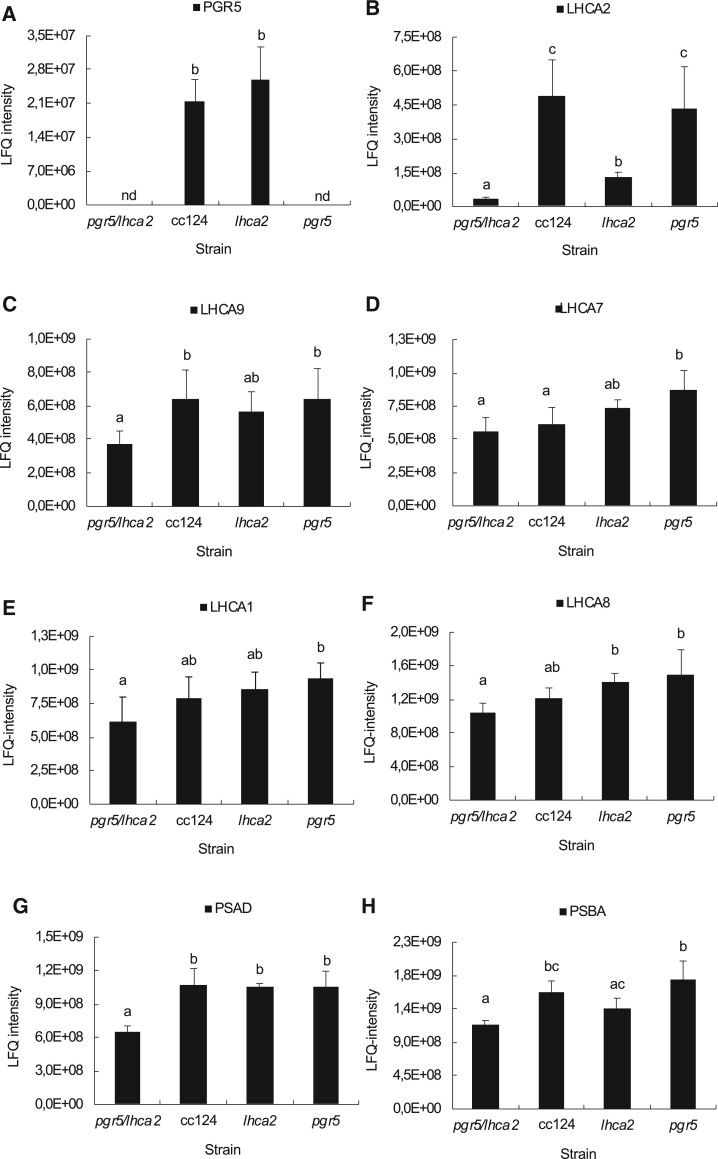
LFQ of PGR5, LHCA2, LHCA9, LHCA7, LHCA1, LHCA8, PSAD, and PSBA proteins in *Chlamydomonas* WT cc124, single mutants *lhca2, pgr5*, and double mutant *pgr5/lhca2*. A, PGR5 protein abundance; (B–F) LHCI proteins LHCA2, LHCA9, LHCA7, LHCA1, and LHCA8, respectively. G and H, photosystems I and II proteins PSAD and PSBA, respectively. All strains were grown in TAP medium at 25°C in normal light conditions with light intensities in 60 µmol photons m^−2^ s^−1^. LFQ intensities of PGR5, LHCA2, LHCA9, LHCA1, LHCA8, PSAD, and PSBA protein abundances are expressed as means (±standard deviation (sd)) from four independent biological replicates. Statistical significance was based on one-way ANOVA with different letters (a–c) indicating significant differences (Benjiamini–Hochberg corrected FDR < 0.05). The abbreviation nd means that proteins were not detected.

**Figure 2:**
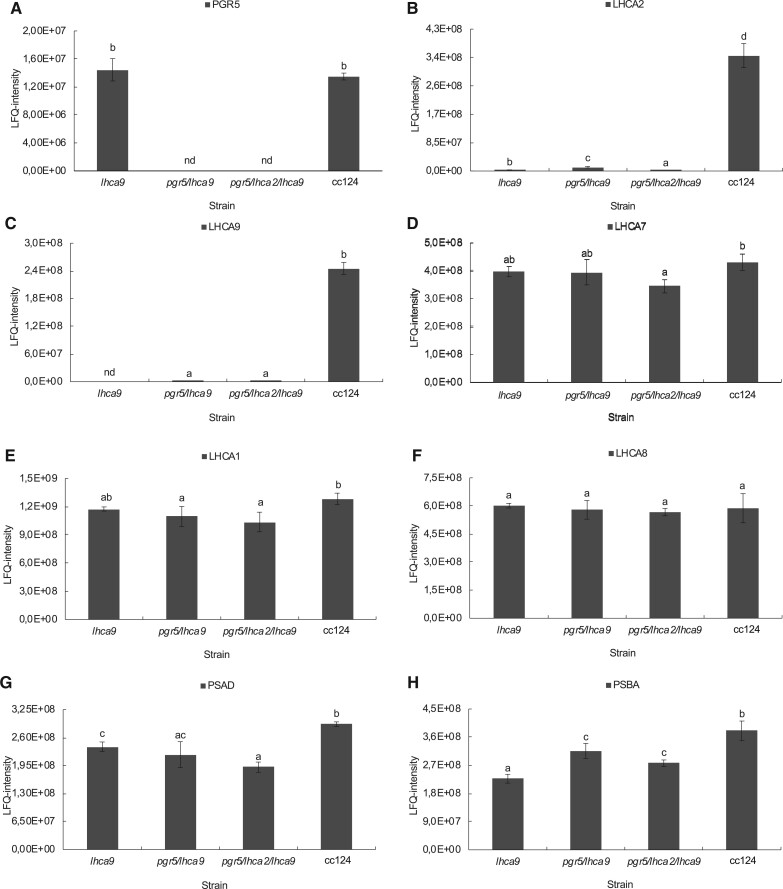
LFQ of PGR5, LHCA2, LHCA9, LHCA7, LHCA1, LHCA8, PSAD, and PSBA proteins in Chlamydomonas WT cc124, single mutants *pgr5* and *lhca9*, double mutant *pgr5/lhca9*, and triple mutant *pgr5/lhca2/lhca9*. A, Protein abundance of PGR5; (B–F) LHCI proteins LHCA2, LHCA9, LHCA7, LHCA1, and LHCA8, respectively. G and H, PSAD and PSBA are one of PSI and PSII proteins, respectively. All strains were grown in TAP medium at 25°C under normal light conditions with light intensities in the range of 60 µmol photons m^−2^ s^−1^. The LFQ intensities of PGR5, LHCA2, LHCA9, LHCA1, LHCA8, PSAD, and PSBA are presented as means (±sd) from at least three independent biological replicates. The abbreviation nd means that proteins were not detected. The letters a–d indicate statistically significant difference with Benjiamini–Hochberg corrected FDR < 0.05.

To test the genetic interaction between *pgr5* (Johnson et al., 2014) and *lhca2* and *lhca9* (Li et al., 2016)*, lhca2, lhca9*, and *pgr5* single mutants were genetically back-crossed three times using WT cc124/cc125 cells. After successful backcrosses, *pgr5/lhca2* and *pgr5/lhca9* double and *pgr5/lhca2/lhca9* triple mutants were generated by additional rounds of genetic crossings. To verify the mutant status, all strains were analyzed by mass spectrometry-based peptide and protein quantification. According to label-free quantification (LFQ), PGR5 was absent in the single *pgr5* as well as in the double and triple mutants. LHCA2 protein was found to be five-fold diminished in the single *lhca2* mutant as compared to WT cc124, while the LHCA2 protein was nearly absent in the double and triple mutants. The presence of LHCA2 in the *lhca2* insertion mutant can be rationalized as the DNA insertion in the *lhca2* gene is located in an intron. Furthermore, other LHCA (LHCA9, LHCA7, LHCA1, and LHCA8), PSI (PSAD), and PSII (PSBA) polypeptides were quantified by LFQ. It appeared that these proteins were rather diminished in the *pgr5/lhca2* double mutant. For LHCA9, PSAD, and PSBA, these differences were significant as compared to WT cc124 (Figure 1, C, G, and H). PSAD and PSBA were also less abundant in *lhca9, pgr5/lhca9* double and *pgr5/lhca2/lhca9* triple mutants (Figure 2, G and H). Mass spectrometric LFQ analyses confirmed that *lhca9, pgr5/lhca9*, and *pgr5/lhca2/lhca9* were deficient in LHCA9 and revealed that, besides LHCA9, LHCA2 was also absent in *lhca9* and *pgr5/lhca9* (see Figure 2). This indicates that LHCA9 was present in the absence of LHCA2, but that LHCA2 required LHCA9 for accumulation.

### Hydrogen photo-production of *lhca2*, *lhca9*, and *pgr5* single, double, and triple mutant strains

**Figure 3:**
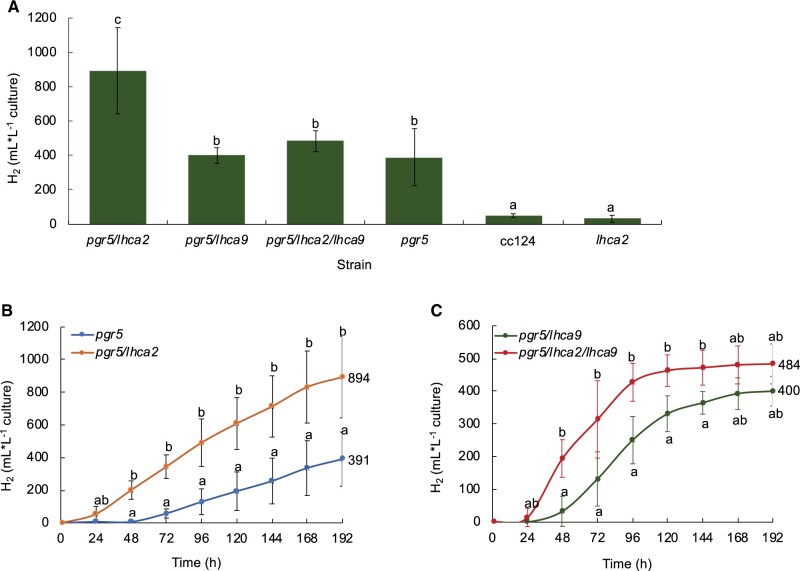
Long-term hydrogen (H_2_) production of WT cc124, *pgr5, lhca2*, *pgr5/lhca2*, *pgr5/lhca9*, and *pgr5/lhca2/lhca9* mutants in 500-mL Schott bottles. A, Total H_2_ volume produced by the mutants on WT cc124 background. B and C, Comparison of time course H_2_ production between double mutant *pgr5/lhca2* and single mutant *pgr5*, and double mutant *pgr5/lhca9* and triple mutant *pgr5/lhca2/lhca9*, respectively. The cultures were grown at 15 mg Chl L^−1^ in 500 mL Schott bottles with one-side illumination (60 µmol photons m^−2^ s^−1^). H_2_ production was measured every 24 h until 192 h under TAP-S medium. Data are expressed as mean (±sd) of 3 ≤ *n* ≤ 5 biological replicates. Statistical significance (*P* ≤ 0.05) based on one-way ANOVA with post-hoc test Turkey (A), Student’s test (*t* test), and Mann–Whitney U for (B) and (C) with different letters indicating significant difference.

**Figure 4:**
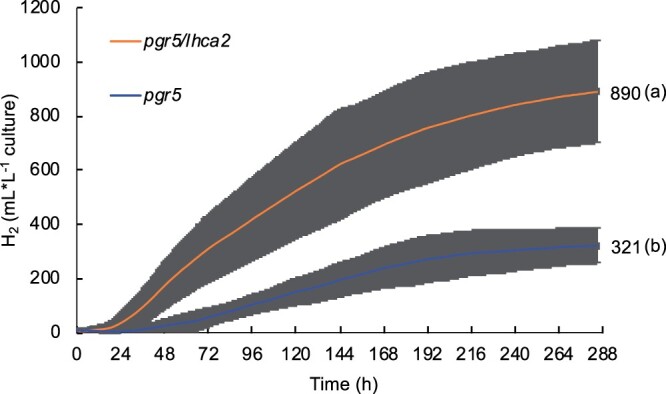
Long-term hydrogen (H_2_) production in 1L BlueSens gas measuring system. H_2_ evolution of *pgr5* single mutant and *pgr5/lhca2* double mutants. Total H_2_ production by single mutant *pgr5* and double mutant *pgr5/lhca2* is shown. The values are expressed as mean of 3 ≤ *n* ≤ 5 independent replicates (± sd). The cultures were started at 15 mg Chl L^−1^ and H_2_ production were measured over 285 h with continuous light 60 µmol photons m^−2^ s^−1^ under four sides illumination. Statistical significance (*P* ≤ 0.05) according to Student’s test (*t* test) with different letters indicating significant difference.

As *pgr5* is a very potent H_2_ producer under anoxia (Steinbeck et al., 2015; Khosravitabar and Hippler, 2019; Nagy et al., 2021), we questioned whether H_2_ photo-production is altered in the double and triple mutants. Therefore, after growth under photo-heterotrophic conditions, mutant strains as well as WT cc124 were shifted to sulfur deficiency to measure their capacity to evolve H_2_. Continuous H_2_ photo-production was measured with two independent measuring systems (Figures 3 and 4). The first approach used sealed 500-mL flasks with a gas collection apparatus similar to that previously reported (Figure 3, A–C;Steinbeck et al., 2015). The total H_2_ gas production of the different strains in the sealed 500-mL flasks is shown in Figure 3A. WT cc124 and *lhca2* evolved <100 mL of gas in the course of 8 d, while *pgr5*, *pgr5/lhca9*, *pgr5/lhca2/lhca9*, and *pgr5/lhca2* produced significantly more with averaged values of 391, 400, 484, and 894 mL H_2_ per L culture, respectively. In this setting, *pgr5/lhca2* produced the most H_2_, which was significantly different as compared to the other strains (Figure 3B). The H_2_ photo-production in *pgr5/lhca2* advanced fast and reached 200 mL of H_2_ after only 2 d. This trend was also observed in *pgr5/lhca2/lhca9* (Figure 3C). In both strains, H_2_ photo-production was significantly more advanced from 48 h to at least 144 h as compared to *pgr5* and *pgr5/lhca9*. Notably, *pgr5* and *pgr5/lhca9* only started to produce H_2_ after 48 h (cf. Figure 3, B and C). The H_2_ production in *pgr5*, *pgr5/lhca9*, and *pgr5/lhca2/lhca9* mutant strains was not significantly different. It is of note that the *pgr5/lhca9* as well as *pgr5/lhca2/lhca9* slowed down in producing H_2_ at 96 h, which is earlier than in *pgr5* and *pgr5/lhca2* (Figure 3, B and C). In the second approach, we took advantage of a BlueSens gas measuring system (BluesSens GmbH, Herten, Germany). H_2_ production from 1 L of culture was measured under constant stirring in a gas tight glass fermenter that was equipped with three gas sensors (H_2_, O_2_, CO_2_, BluesSens GmbH, Herten, Germany) constantly recording the composition of the headspace (in Vol%) (Figure 4, see also Supplemental Figure S1). In the BlueSens setup (Figure 4), measurements were focused on *pgr5* and *pgr5/lhca2*. In a timeframe of 12 d, *pgr5* and *pgr5/lhca2* produced on average 321- and 890-mL H_2_ per L culture, respectively (Figure 4). Thus, in the two distinct operating systems, the *pgr5/lhca2* strain produced significantly more hydrogen than *pgr5* (Figures 3 and 4).

### Membrane inlet mass spectrometry of *pgr5/lhca2* and *pgr5* mutant strains

**Figure 5:**
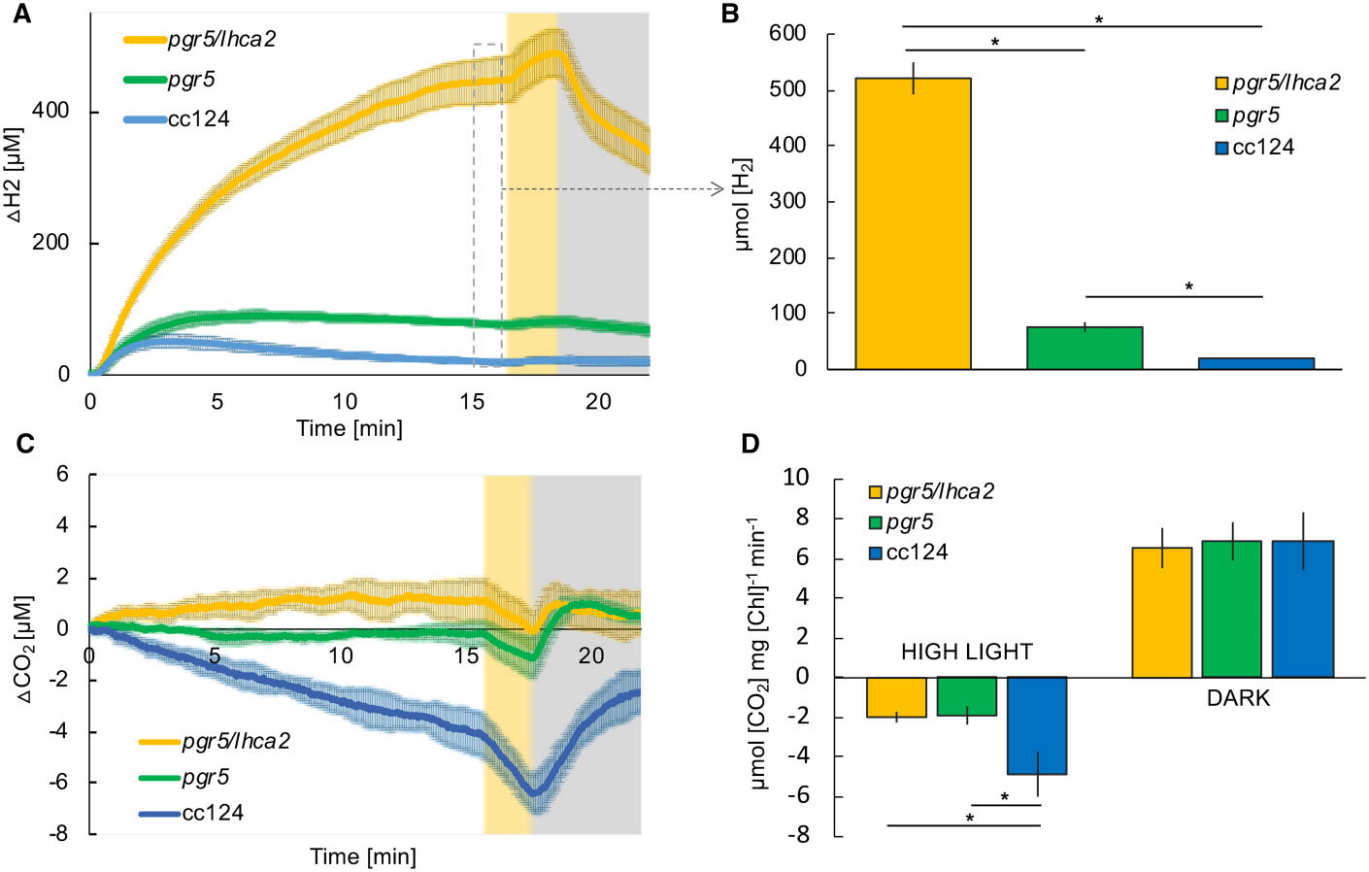
Short-term hydrogen (H_2_) production and CO_2_ assimilation and release. Short-term kinetics of dissolved H_2_ (A) and CO_2_ (C) measured by MIMS. Cc124, *pgr5/lhca2*, and *pgr5* cells at a concentration of 15 µg Chl mL^−1^ were incubated in the dark for 2 h, after which they were exposed to 16 min of illumination (370 µmol photons m^−2^ s^−1^; white background) followed by 2 min of high light (2,500 µmol photons m^−2^ s^−1^; yellow background). B, Absolute values of the H_2_ [µmol] accumulated after 16 min of continuous illumination, shown as bars graph. D, The rates of CO_2_ assimilation in high light and afterwards its release in the dark (yellow and gray background, respectively). Mean values and sd bars are shown (*n* = 3). For B and D, *pgr5*/*lhca2* was found statistically distinct from *pgr5* and cc124 where indicated with the stars, analyzed by two-tailed *t* test (*P* < .05).

To assess H_2_ photo-production in *pgr5* and *pgr5/lhca2* production in different scenario, we measured H_2_ production as well as CO_2_ uptake under ambient conditions using membrane inlet mass spectrometry (MIMS) (Figure 5). Cells of *pgr5/lhca2*, and *pgr5* mutant strains as well as WT cc124 were grown under photo-heterotrophic conditions, dark-adapted for 1 h and shifted to a light intensity of 370-µmol photons m^−2^ s^−1^ for 16 min, followed by a strong light pulse of 2,500-µmol photons m^−2^ s^−1^ for 2 min and another dark phase. In this experimental setup, H_2_ production after light onset and light pulse was by far highest in *pgr5/lhca2* as compared to the two other strains (Figure 5A). After 16 min, *pgr5/lhca2* had accumulated >500 µM of H_2_, while *pgr5* and WT cc124 accumulated only about 80 and 10 µM of H_2_, respectively (Figure 5B). This revealed that under such particular conditions *pgr5/lhca2* is indeed a potent H_2_ producer. In contrast, CO_2_ uptake started in the WT cc124 immediately after the onset of light and was boosted by the strong light pulse. In *pgr5*, only minor net CO_2_ uptake was observed after the light was turned on (Figure 5C). Yet, upon the strong light pulse, CO_2_ uptake is seen but to a lower extent as compared to WT. In *pgr5/lhca2*, the onset of light resulted rather in a CO_2_ release, while the strong light pulse caused a miniscule CO_2_ uptake in this strain. Rates of CO_2_ assimilation in high light and CO_2_ release in the dark were determined (Figure 5D) and revealed that in the light CO_2_ uptake was significantly faster in the WT as compared to *pgr5* and *pgr5/lhca2*, while CO_2_ release rates were similar between the strains.

### Assessing photosynthetic electron transfer capacities

**Figure 6:**
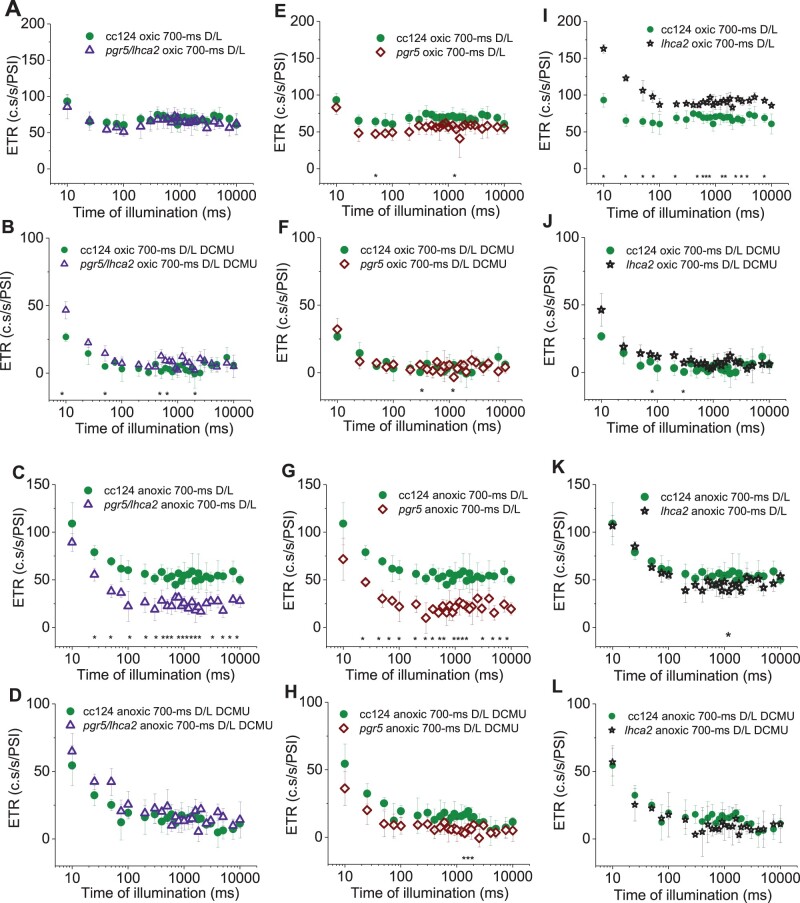
The second consecutive ETR measurement via the dark pulse method is shown for the strains *pgr5/lhca2*, *pgr5*, *lhca2*, and WT cc124. The ETR was produced after a 700-ms short dark-phase and is expressed as charge separations.s^-1^.PSI^-1^ (c.s/s/PSI), after the measurement presented in Supplemental Figure S3 (for reference of the measurement routine, see also Supplemental Figure S2). A, E, and I, ETR in oxic condition (B), (F), (J), ETR in oxic conditions and in the presence of DCMU. C, G, and K, ETR in anoxic conditions, (D), (H), (L) ETR in anoxic conditions and in the presence of DCMU. Each time point is an average of at least three biological replicates (±sd) with statistical comparisons analyzed by Student’s *t* test (^*^P ≤ 0.05).

**Figure 7:**
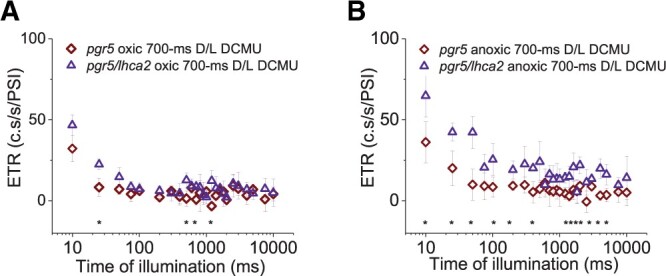
The second consecutive electron transfer rate (ETR) measurement via the dark pulse method is shown for the strains *pgr5/lhca2* and *pgr5* after a 700-ms dark phase. The ETRs, expressed as charge separations.s^-1^.PSI^-1^ (c.s/s/PSI), were produced in oxic (A) and anoxic conditions (B) after DCMU treatment, as presented in Figure 6 (for reference of the measurement routine, see also Supplemental Figure S2). Each time point is an average of at least three biological replicates (±sd) with statistical comparisons analyzed by Student’s *t* test (^*^P ≤ 0.05).

**Figure 8:**
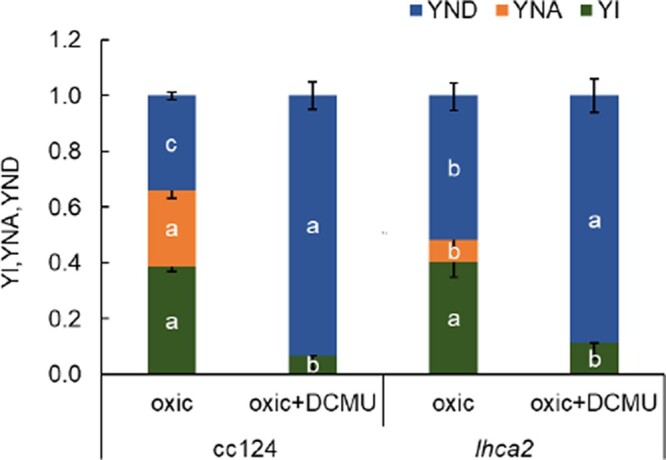
P700 deconvolution between WT cc124 and lhca2 under oxic conditions. The photo-oxidizable P700 fractions (YI), nonoxidizable P700 because of acceptor side limitation (YNA), and pre-oxidized P700 due to donor-side limitation (YND) were examined before and after the addition of DCMU for three biological replicates. By employing a one-way ANOVA with Turkey post hoc test (*P* < 0.05), the data for each group are presented as an average (±sd) with different letters indicating statistical significance.

In a next set of experiments, we explored the photosynthetic electron transfer capacity in the WT and mutant strains under oxic and anoxic photo-autotrophic conditions. We measured total electron transfer rates (ETRs) in cells, which were grown at low light intensities at 7- to 15-µmol photons m^−2^ s^−1^. For determining the ETR (see Materials and methods), the electrochromic shift (ECS) signals were measured using a Joliot-type spectrophotometer. In brief, all absorption changes were normalized to the ECS ΔI/I signals (520–546 nm) produced after a saturating laser flash in the presence of 1-mM hydroxylamine (HA) and 10-µM 3-(3,4-dichlorophenyl)-1,1-dimethylurea (DCMU). Thus, the flash-induced rapid ECS describes the density of active PSI centers in PSII-inhibited HA/DCMU samples (measured as 1 charge separation.PSI^−1^). The ECS was also deconvoluted using the dark pulse method (reviewed in Joliot and Joliot, 2002; Bailleul et al., 2010; Nawrocki et al., 2019) and ETRs were calculated (the electron transfer measurement scheme is shown in Supplemental Figure S2). The ETR are shown for a 10-s illumination period after 20 min of dark adaptation (Supplemental Figure S3). As 20 min of darkness likely affected the redox state of the PQ pool due to reduction via Nda2 (Desplats et al., 2009), an additional 700-ms short dark period was introduced, which was followed by another 10-s illumination period and measurement of ETRs (Figure 6). The latter measurements were a better representation for electrons solely stemming from photosynthesis-driven electron transfer. The ETR measured after 20 min of dark adaption Supplemental Figure S3 as well as after the second short 700-ms dark phase (Figure 6), are characterized by an initial 300-ms phase of photosynthetic induction where rates were first high and then declined, followed the establishment of a steady state. Overall, initial ETRs were more advanced after 20 min of dark adaption. Notably, ETRs in the *lhca2* mutant were significantly higher in oxic conditions (part I in Supplemental Figure S3; Figure 6) as compared to WT cc124, *pgr5*, and *pgr5/lhca2* (respective parts E and A in Supplemental Figure S3; Figure 6). This was also true after DCMU treatment (parts B and F in Supplemental Figure S3; Figure 6) although the *lhca2* strain outperformed WT cc124 significantly only at two time points after long and short dark adaptation (Supplemental Figure S3J; Figure 6J). Under anoxic conditions, ETRs in *pgr5* and *pgr5/lhca2* were significantly lower in steady state after the 20-min dark adaptation (Supplemental Figure S3, C and G), while after the second short dark phase, these differences in comparison to WT cc124 were even evident in the initial photosynthetic induction phase (Figure 6, C and G). DCMU treatment did only impact ETR in *pgr5*, where differences to WT cc124 were observed at two time points (Figure 6H). Interestingly, under anoxia, the ETR in *pgr5/lhca2* in the presence of DCMU was significantly faster than in *pgr5* (seen for 13 time points). Under oxic conditions, this significant difference was observed for four time points (Figure 7, A and B). Notably, the ECS measurements provided evidence that the overall photosynthetic electron transfer in *lhca2* was more advanced than in WT cc124 under oxic conditions. This was also evident for ETR in the presence of DCMU in oxic conditions, which has been already observed for *lhca2* with regard to WT (Steinbeck et al., 2018). In addition, we measured the redox state of P700 in *lhca2* and WT cc124 under oxic conditions according to an established protocol based on the method by (Klughammer and Schreiber, 1994) (Figure 8). While YI (the photo-oxidizable P700 fraction after 10 s in the light) was similar between *lhca2* and WT cc124, acceptor side (YNA) and donor side limitation (YND) were significantly different. In this setting, YNA was smaller and YND was larger in *lhca2* as compared to WT cc124. The P700 redox parameters are interdependent and considering the higher ETRs in oxic *lhca2* versus WT cc124, detection of an elevated YND was not limiting the overall electron transfer under these conditions in the presence of oxidized PSI electron acceptors pools.

As indicated above, ETR in *pgr5/lhca2* in the presence of DCMU was significantly increased under anoxia, denoting that the absence of LHCA2 seemed to rescue the ETR in the presence of DCMU in the *pgr5* mutant. This suggests that LHCA2 might act as a suppressor of PGR5 function in electron transfer regulation under conditions, where PSII activity is blocked. We also measured the redox state of P700 in all four strains under anoxia (Supplemental Figure S4). As previously observed (Buchert et al., 2020), YNA and YND were significantly different in WT cc124 and *prg5*, where WT had a pronounced YNA, which was considerably smaller in *pgr5*. On the other hand, YND was significantly larger in *pgr5* than in WT. Here, in regard to YNA and YND, *lhca2* and *pgr5/lhca2* followed WT and *pgr5*, respectively. Concerning the DCMU-treated samples, no significant differences in YI and YND were observed in the strains. YND was similar in *lhca2* versus WT cc124 in the absence of DCMU, yet considerably increased in *pgr5/lhca2* and *pgr5*. As mentioned, high YND allowed fast ETR in *lhca2* when PSI electron acceptor pools were not saturated under oxic conditions. In contrast, the total ETRs in anoxic cells were significantly diminished in the high-YND strains *pgr5/lhca2* and *pgr5*, suggesting that detection of elevated YND becomes limiting for overall electron transfer once cells are in a reducing environment which otherwise favors YNA.

We also measured ETR in *lhca9* as well as *pgr5/lhca9*. Interestingly, the ECS measurements revealed that, as seen in *lhca2*, ETR in *lhca9* is significantly faster than in WT cc124 under oxic conditions (Supplemental Figure S5). In contrast to *lhca2*, ETR in *lhca9* was not significantly different under anoxia and DCMU after the second short dark phase as compared to WT cc124 and rates were already similar to the *pgr5* strain after 100 ms. In this regard, LHCA9 did not seem to suppress the PGR5 ETR phenotype under anoxia and DCMU.

## Discussion

In this work, we provide evidence that both the *lhca2* and *lhca9* mutants revealed high photosynthetic ETR in oxic conditions, that LHCA2 impacts the *pgr5* phenotype and that *pgr5/lhca2* is a potent H_2_ producer. In addition, *pgr5*/*lhca2* and *pgr5*/*lhca9* revealed significantly different H_2_ photo-production kinetics, indicating that the absence of LHCA2 or LHCA9 impact H_2_ photo-production independently, pointing to distinct regulatory capacities.

### Efficient hydrogen production in *pgr5/lhca2*

The *pgr5* mutant has been described as an effective H_2_ producer with maximal production of about 800-mL H_2_ per L algal culture (Steinbeck et al., 2015). To study the genetic interaction between the *pgr5* and *lhca2* and *lhca9* mutations, all mutants were back-crossed in the cc124/125 genetic backgrounds, and double mutants as well as the triple mutant were generated. In the altered genetic background, the *pgr5* mutant produced between 330 and 390 mL H_2_. This is significantly less than in the T222 parental background (Steinbeck et al., 2015), indicating that the genetic background influences the *pgr5* phenotype. Yet, H_2_ photo-production was still more than seven-fold higher as compared to the respective WT, as shown before (Steinbeck et al., 2015) (Figure 3A). The addition of the *lhca2* mutation to *pgr5* boosted H_2_ photo-production back to almost 900 mL of H_2_ in both gas measurement systems (Figures 3 and 4), being more than 17-fold higher as compared to WT. This 17-fold increase in H_2_ photo-production is a substantial improvement, as compared to other described mutants (Toth and Yacoby, 2019). Moreover, the MIMS measurements under ambient conditions revealed, that *pgr5/lhca2* outperformed *pgr5* significantly (Figure 5), despite ETR in *pgr5/lhca2* and *pgr5* being very similar, yet slower as compared to WT (Figure 6). The slower ETR rates can be likely explained with the finding that electron transfer in a *pgr5* genetic background is donor side limited as the turn-over of the Cyt *b_6_f* complex is slowed down in *pgr5* (Buchert et al., 2020).

### PSI remodeling may result in stromal electron rerouting toward hydrogenase

How can the improved H_2_ production of *pgr5/lhca2* be rationalized? Under oxic condition, the ETR in *lhca2* and *lhca9* was enhanced, but not under anoxia, where H_2_ was produced. Under anoxia, hydrogenase competes successfully for PSI electrons that originate from H_2_O via LEF or from CEF processes. Although H_2_ photo-production is strongly PSII-dependent (Antal et al., 2003; Kosourov et al., 2003; Fouchard et al., 2005; Hemschemeier et al., 2008), CEF contributes as well because it limits electron flow into the vast stromal space. Thereby, controlling the local transfer pathway of an electron by cycling between PSI and Cyt *b_6_f* may favor its interception by hydrogenase (Yacoby et al., 2011; Eilenberg et al., 2016). Importantly, the entanglement of CEF and H_2_ production under physiological conditions is presently unknown, owing to the methodical bottleneck of assessing CEF without inhibiting PSII activity. However, *pgr5/lhca2* improved ETR in regard to *pgr5* under anoxia in the presence of DCMU (Figure 7), suggesting CEF processes in the highly potent H_2_ producer *pgr5/lhca2*. In fact, ETR in the presence of DCMU is linked to the capacity of CEF, as it is solely driven by electron transfer between PSI and the cyt *b_6_f* complex. This is in line with increased CEF in *lhca2* under oxic DCMU conditions (Steinbeck et al., 2018). It has been suggested that the absence of LHCA2 and LHCA9 might promote CEF by the formation of a PSI–Cyt *b_6_f* complex supercomplex, according to the low-resolution structural data devoid of LHCA2 and LHCA9 (Steinbeck et al., 2018). Interestingly, our data also indicate that the total ETR, that is, the combination of LEF together with CEF, was transiently enhanced in the absence of LHCA2 and LHCA9 when hydrogenase was not a competent electron sink due to oxygen in the sample (Supplemental Figures S3, 5, and 6; Figure 6). A possible enhancement of CEF contributions to the total ETR in *lhca2* is also in line with an increased donor side limitation (Figure 8), as CEF would further acidify the lumen resulting in an augmented photosynthetic control (Stiehl and Witt, 1969).

As shown, LHCA2 did not accumulate in the absence of LHCA9, while LHCA9 was present in the absence of LHCA2 (Figures 1 and 2). In agreement, PSI particles isolated from a *lhca2* mutant contained LHCA9, but PSI particles isolated from a LHCA9 deficient strain did not possess LHCA2 (Naschberger et al., 2021). A recent cryo-electron microscopy structural study identified two LHCA9 copies as the key elements for the dimerization of PSI from *C. reinhardtii* (Naschberger et al., 2021). In the dimeric PSI structure, LHCA2 is absent. Thus, the absence of LHCA2 could promote the formation of PSI-dimers, while the absence of LHCA2 and LHCA9 could support the formation of a PSI–cyt *b_6_f* complex supercomplex (Steinbeck et al., 2018). The formation of a state II PSI–LHCI–LHCII complex, where two LHCII trimers are linked per PSI (Huang et al., 2021; Pan et al., 2021), is hampered in the absence of LHCA2 and LHCA9 (Naschberger et al., 2021). Yet, under anoxia, where State II is more likely, ETR in *lhca2* and *lhca9* was not increased in the same way as under oxic conditions (Supplemental Figure S3, 5, and 6; Figure 6). This makes changes in state transitions and/or light-harvesting antenna composition not a likely reason for differences in LEF and amount H_2_ produced. The increase of CEF in anoxia and DCMU in *pgr5/lhca2*, however, could be related to PSI remodeling and the potential formation of PSI-dimers, which would be favored in the absence of LHCA2. Moreover, the increased robustness of *pgr5/lhca2* in H_2_ production as compared to *pgr5/lhca9* and *pgr5/lhca2/lhca9* might be linked to PSI-remodeling. It also indicates that the increase in H_2_ production in *pgr5/lhca2* cannot simply be explained by decreased light-harvesting capacity as *lhca9* is depleted both in LHCA2 and LHCA9. Previously, it has been reported that engineered strains with lower light-harvesting protein content revealed higher H_2_ production (Oey et al., 2013). Thus, the fast onset in H_2_ production in *pgr5/lhca2* and *pgr5/lhca2/lhca9* (Figures 3 and 4) could also be related to PSI-remodeling. It is tempting to speculate that the absence of LHCA2 and/or LHCA9 establishes structural remodeling of PSI such as endorsing PSI–cyt *b_6_f* supercomplex formation or promoting dimeric PSI related supercomplexes, that in turn might influence LEF/CEF capacity and/or electron partitioning at the PSI acceptor side.

Thus, enhanced hydrogen production in *pgr5/lhca2* might be related to PSI-dependent structural remodeling that would promote overall electron transfer and particularly electron transfer toward hydrogenase. The MIMS data support this view, as *pgr5/lhca2* released CO_2_ in the light, while H_2_ production increased steadily in the light resulting in an accumulation of 500 µM of H_2_ in 16 min, corresponding to 62.5-µmol H_2 _mg^−1^ chl h^−1^ at 370 µmol photons m^−2^ s^−1^. Milrad et al. (2018), revealed that in a 2-min MIMS experiment under conditions when Calvin cycle is not active, WT algae may produce up to 150-µmol H_2_ mg^−1^ chl h^−1^ at 370 µmol m^−2^s^−1^, which drops steeply upon activation of CO_2_ fixation. In *pgr5/lhca2*, a high rate of H_2_ production could be maintained for 16 min, while net CO_2_ fixation was absent, further supporting the view that Calvin Cycle is competitive to H_2_ production as suggested (Milrad et al., 2018). The less efficient rate of CO_2_ assimilation (Figure 5) and high O_2_ uptake as observed in *pgr5* (Steinbeck et al., 2015), indeed suggest that, despite similar photosynthetic ETR in *pgr5/lhca2* as compared to WT (Supplemental Figure S3; Figure 6A), electrons are predominately transferred to hydrogenase or exploited for O_2_ uptake in *pgr5/lhca2*, whereas in WT, more electrons are utilized for CO_2_ fixation (Figure 5). In *pgr5*, which has a pronounced respiration (Steinbeck et al., 2015), rate of CO_2_ assimilation was lower, as in WT, and comparable with *pgr5/lhca2*. Increased H_2_ production in *pgr5* versus WT would also indicate that more electrons are dedicated to H_2_ production, yet less as observed in *pgr5/lhca2*. A scenario where photosynthetic electrons in *pgr5/lhca2* are more efficiently subjected to hydrogenase might also explain the high H_2_ production under sulfur deficiency (Figures 3 and 4). In *C. reinhardtii*, the FNR which produces NADPH required for CO_2_ fixation is 70-fold more abundant than hydrogenase (Nikolova et al., 2018). Thus, it is tempting to speculate that PSI-dimer formation in *pgr5/lhca2* might support H_2_ production via recruiting hydrogenase to PSI and/or by displacing FNR (see below). The fact that more CEF is observed in *pgr5/lhca2* (Figures 6, B and 7) as discussed above, is not necessarily a discrepancy but possibly reflects PSI structural remodeling which at the same time supports electron transfer toward hydrogenase.

### PGR5 and LHCA2 functions are interrelated

Another open question is how LHCA2 could impact PGR5 function? It has been suggested that PGR5 is required for efficient Q-cycle and PSI-dependent stromal electron input into the cyt *b_6_f* complex under anoxia (Buchert et al., 2020). At the same time, PGR5 impacts binding of FNR to thylakoid membranes in *C. reinhardtii* (Mosebach et al., 2017). A delocalization of FNR from the *pgr5* PSI stromal interface could promote electron flow into the stroma, becoming inaccessible for the input into the cyt *b_6_f* complex under anoxia. This *pgr5* defect may be mitigated by the concomitant absence of LHCA2 which reveals the PSI docking interface for the cyt *b_6_f* complex (Steinbeck et al., 2018), thus limiting the drain of PSI electrons into the stromal space. A delocalization of FNR from PSI may also be another reason for more efficient electron transfer to hydrogenase in *pgr5/lhca2*, as discussed above. Thus, the structural remodeling of stromal features, facilitation of PSI–cyt *b_6_f* assemblies, and/or oligomerization states of PSI might distinctly support CEF that is independent of PGR5, thereby suppressing the PGR5 phenotype in regard to CEF (Figure 7). It is interesting to note, that the absence of *lhca2* only impacts the *pgr5* phenotype when PSII activity is lacking (Supplemental Figure S3; Figures 6 and 7), whereas in the presence of PSII activity the *pgr5* phenotype dominates in *pgr5/lhca2*. Apart from redox poising the PQ pool, PSII activity contributes significantly to membrane energization required for efficient cyt *b_6_f* and ATP synthase turnovers (Junge, 1970; Junge et al., 1970; Barbagallo et al., 2000) as well as build-up of photosynthetic control (Stiehl and Witt, 1969). Moreover, PSII also fuels hydrogenase (see above). Certainly, by inhibiting PSII a new energization state of the redox-controlled system is obtained in the light. Thus, it is tempting to speculate why the absence of LHCA2 impacts the bottleneck imposed by *pgr5* when the LEF-decoupled system is examined under “CEF only” conditions. It might be related to a DCMU-poised shift in electron sink capacities, the membrane energization state and/or PSI donor side limitation. Notably, *lhca2* increased donor side limitation under oxic conditions when PSII was active, likely via an increase of CEF (Figure 8). In addition, depletion of LHCA2 slightly diminished donor side limitation in *pgr5* under anoxia, which is in line with higher CEF as observed in *pgr5/lhca2* in the presence of DCMU (Figure 7) and might indicate that stromal electron intake is partially restored, as speculated above, in the absence of PGR5 and LHCA2. Together, this would support that *lhca2* impacts PSI donor side limitation.

In conclusion, our work identified the *pgr5/lhca2* mutant as a very efficient photo-H_2_ producer. At the same time, it relates hydrogen production as well as photosynthetic electron transfer to PSI remodeling processes and reveals that H_2_ production is controlled by electron partitioning at the PSI acceptor side. We suggest that the absence of LHCA2 enhances photo-H_2_ production via improved binding of hydrogenase to PSI and/or electron transfer toward hydrogenase via FDX1 after its photo-reduction by PSI (Supplemental Figure S7). As pgr5/*lhca2* and pgr5/*lhca9* revealed significantly different H_2_ production kinetics, LHCA2 and LHCA9 are probably involved in distinct regulatory tasks.

## Materials and methods

### Strains and maintenance conditions

*Chlamydomonas reinhardtii* single mutants *lhca2* LMJ.RY 0402109691 and *lhca9* LHJ.RY04022392761 were collected from Chlamydomonas Library project (https://www.chlamylibrary.org) (Li et al., 2016, 2019), and *pgr5* was obtained from T222 background (Johnson et al., 2014). The mutant strains *lhca2, lhca9*, and *pgr5* were backcrossed with WT cc124^−^/cc125^+^ three times independently to make the genetic background comparable. The backcrosses were verified with mating type primers MID_Fw (5′-ATGGCCTGTTTCTTAGC-3′), MID_Rev (5′-CTACATGTGTTTCTTGACG-3′), FUS1_Fw (5′-ATGCCTATCTTTCTCATTCT-3′), and FUS1_Rev (5′-GCAAAATACACGTCTGGAAG -3′). The offspring of *lhca2, lha9*, and *pgr5* were analyzed by the following primer LHCA2 _Rev (5′-ACA CAA ACA CAA GGG GAA GC-3′), LHCA2_Fw (5′-GTC ATC TTT CAC CCG CAA AT-3′), LHCA9_ Rev (5′-CTC AGC CCT TCA GCG ATC CT-3′), LHCA9_Fw (5′-GAC CGC TTT GTG TTA CGC TC-3′) PGR5 _Rev (5′-AAG CCC AGC TTC TCG CCG TT-3′), and PGR5_Fw (5′-TCC AAG CCC GTT GTT GGC GT-3′), respectively. As the locus of *lhca2* and *lhca9* mutant was disrupted with the insertion CIB1 cassette, conferring resistance to paromomycin, the presence of the cassette was assessed by insertion primer OMJ 944 (5′-GAC GTT ACA CGA CAC CCT TG-3′) with LHCA2_Fw (5′-GTC ATC TTT CAC CCG CAA AT-3′) and LHCA9_Fw (5′-AGA TCC CCT GTT ACA TCC CC-3′), respectively. Finally, *pgr5/lhca2* and *pgr5/lhca9* double mutants were created via mating *pgr5* with *lhca2* or *lhca9* mutant, while triple mutant *pgr5/lhca2/lhca9* was generated via crossing *pgr5, lhca2*, and *lhca9* together. The strains were cultivated on TAP agar medium with 1.5% (w/v) agar, pH 7.0 in constant low light intensities from 20 to 30 µmol photon m^**−**2^s^**−**1^ at 25°C in the alga growth chamber.

### Growth conditions

For proteomics analysis, cells for four biological replicates were inoculated in Erlenmeyer flasks containing 20 mL in Tris-acetate phosphate (TAP) medium, pH 7, 25°C under continuous illumination from 50 to 60 µmol photon m^**−**2^s^**−**1^, shaking at 120 rpm. Twenty-four hours before harvesting, the cells were diluted with a fresh TAP medium to harvest them in the exponential growth phase.

For in vivo hydrogen production, cells were grown initially in 50 mL of standard medium TAP in continuous light intensities ranging from 20 to 30 µmol photon m^**−**2^s^**−**1^, shaking at 120 rpm. After 3 to 4 d, the cultures were upscaled to 200 mL, 1 L, or 2 L cultures in the same medium and growth conditions.

As for ETR, the strains were cultivated in Erlenmeyer flasks (500 mL) contain liquid tris–phosphate medium free acetate (pH 7.0) with sterile air bubbling, light intensities 7–15 µmol photon m^**−**2^s^**−**1^ with 16-h light/8-h dark and placed on constant shaker (120 rpm, 25°C) from 6 to 7 d. Cultures were diluted in fresh TP medium 2 d before measurement.

### Sample preparation and mass spectrometry analysis

In brief, the cells were harvested, removed supernatant (14,000*g*, 5 min, 4°C), and pellets were frozen in liquid nitrogen, stored afterward at −80°C. After extracting the samples in the Lysis buffer, the protein concentrations were determined via the Pierce TM BCA Protein Assay kit instructions (Thermo Scientific, Waltham, MA, USA). Proteins (50 µg/sample) was then digested at least 18 h at 37°C into Trypsin enzyme (PROMEGA, Madison, WI, USA) (protein/enzyme ratio 50:1) according to the Filter Aided Sample Preparation protocol (Wisniewski et al., 2009). The reduction and alkylation were modified by fulfillment in 10 mM Tris (2-carboxyethyl) phosphine and 40-mM chloroacetamide in 8-M urea/100 mM Tris/HCl, pH 8.5 at the same time. For each 5-µg peptide aliquot, StageTips c18 were utilized for sample desalting (Rappsilber et al., 2007), then dried at vacuum centrifugation (Concentrator Plus, Eppendorf) and stored at −80°C. Peptides were soluble in 2% (v/v) acetonitrile/0.05% (v/v) trifluoroacetic acid in Millipore water at concentration of 1 µg/µL in theoretical.

The LC–MS/MS analysis were monitored on an Ultimate 3000 RSLC nano LC System (Thermo Scientific, USA) coupled via nano spray interface to an Q Exactive Plus mass spectrometer (Thermo Scientific, USA). For the raw data, the LFQ was evaluated in MaxQuant 1.6.14.0 (Cox and Mann, 2008). The required false discovery rate (FDR) was set to 1% for both identified peptides and proteins. The database from Chlamydomonas version 5.6 gene models (Joint Genome Institute, www.phytozome.org), combined with mitochondrial and chloroplast protein sequences from NCBI databases BK000554.2 and NC_001638.1. Carbamidomethylation of cysteines were utilized for modification. The primary LFQ data then was imported into Perseus (version 1.6.13.0.2) (Tyanova et al., 2016) for late analysis. Statistics were carried out by one-way ANOVA using Tukey’s range test (FDR 5%) as the post hoc test.

### In vivo hydrogen production

The cells were harvested in the growth phase, washed three times with TAP Sulfur deprivation (TAP-S) medium (2,500*g*, 5 min, 22°C) (Melis et al., 2000) and then resuspended in a sealed 500-mL glass bottle (Schott for mass spectrometer measurements) or a 1 L in gas tight glass fermenter illuminated (BlueSens gas measuring system) in TAP-S medium at the final concentration of chlorophyll 15 mg L^−1^. Each culture was placed on a constant’s stirrer and facing a continuous light intensity at 60–65 µmol photon m^−2^s^−1^ in one side illumination (Schott) or/and four sides illumination (BlueSens). The percentage of hydrogen production was monitored every 24 h by gas chromatography, gas volume was measured by the water level in 100-mL syringe connecting with the glass bottles via needle inserting a tube into the rubber lid seal. For the BlueSens gas measuring system, the fermenter was equipped with three gas sensors (H_2_, O_2_, CO_2_, BlueSens GmbH, Herten, Germany) constantly recording the composition of the headspace (in Vol%). The total volume of gas produced was automatically analyzed every 2 s or/and 5 min with a gas volume counter (BlueV Count) attached via a cold trap (Supplemental Figure S1).

### MIMS analysis

MIMS analysis was performed as described by Liran et al. (2016). For gas exchange measurements, 5 mL of cells at a concentration of 15 μg Chl mL^−1^ in TAP supplemented with HEPES [50 mM] were placed in a cuvette fitted into a metabolic chamber (Optical unit ED-101US/MD, Walz). Following 2-h dark incubation, the cells were exposed to 370 or 2,500 µmol photon m^−2^.s^−1^ of a red actinic light using a Dual-Pulse Amplitude Modulated Fluorometer (DUAL-PAM-100; Heinz Walz Gmbh, Effeltrich, Germany). A standard curve described in (Liran et al., 2016) was used to normalize H_2_, O_2_, and CO_2_ traces. Masses were detected using a 0.5-s dwelling time per mass.

### Measurement of ETR and P700 redox states

For sample preparation, the cells were harvested by centrifugation (4,000*g*, 7 min, 25°C) and chlorophyll concentration was adjusted to 20 µg mL^−1^ in TP medium containing 20% (w/v) Ficoll (PM 400) in open cuvettes. The cells were dark adapted on a shaker set to ∼120 rpm for 20 min prior to measurement. To maintain oxic conditions, samples were mixed regularly every 2 min during measurements. For anoxic conditions, 50-mM glucose, 10-U glucose oxidase, and 30-U catalase were added to each sample cuvette, which were then covered with a layer of mineral oil. Dark incubation of anoxic samples was at least 20 min. For PSII inhibition, each sample was resuspended with 1-mM HA (1 mM) and 10-µM DCMU (Buchert et al., 2020).

ETR calculations were performed by ECS measurements using Joliot type spectrometer devices (for Figures 6 and 7, JTS-10, Biologic, France and for Supplemental Figures S5 and S6, Y JTS-150, Spectrologix, USA). Multi-wavelength measurements were carried out simultaneously with the JTS-150, and the ΔI/I absorption changes in the JTS-10 were measured with white detection pulses in combination with interference filters peaking at 520 and 546 nm (520–546 ECS signals). Photodiodes were protected by BG39 filters (Schott, Germany). The ETR method, measuring stable charge separation rates, is based on the dark pulse protocol (Joliot and Joliot, 2002; Nawrocki et al., 2019). All ECS signals were normalized to one charge separation per PSI, recorded as the ECS signal amplitude in the presence of HA and DCMU that were produced ∼300 µs after a single turnover Nd:YAG laser flash (∼6-ns duration, Minilite II, Continuum). Thus, even when PSII was not inhibited the ECS data was normalized to PSI signals. The cells were dark-adapted for 20 min and experienced two consecutive continuous illumination periods of 10-s actinic light (∼150-µmol photons m^−2^ s^−1^ peaking at 630 nm), spaced by 700-ms darkness (Supplemental Figure S2). Over the course of the 10-s illumination, ECS slopes were recorded before (S_L_) and after the onset of a 5-ms dark interval (S_D_). Contribution of possible charge recombinations in the first millisecond of darkness were avoided by calculation S_D_ after cessation of those electrogenic processes (Nawrocki et al., 2019). Two separate sequences with an offset of the dark pulses were combined in each ETR panel to keep accumulative dark interval artifacts to a minimum. Maximal photochemical rates, plotted as 1 ms of illumination in the ETR panels, were obtained from independent ECS slopes of dark-adapted material during the first 2 ms of actinic illumination. Thus, ECS slopes were only limited by antenna size and actinic light intensity.

Measurements of the P700 redox state were obtained in dark-adapted cells at the end of a 10-s continuous illumination period (∼150-µmol photons m^−2^ s^−1^ peaking at 630 nm). The method is based on a previous protocol (Klughammer and Schreiber, 1994). Multi-wavelength measurements were carried out simultaneously with the JTS-150 and the ΔI/I absorption changes in the JTS-10 were detected with pulses of 700-nm LEDs in combination with 705- and 740-nm interference filters (705–740 P700 signals). Photodiodes were protected by RG695 filters (Schott, Germany). The P700 fractions at the end of continuous illumination period could be divided into pre-oxidized due to donor-side limitation (YND), photo-oxidizable by a 22-ms saturating pulse on top of continuous light (YI) and nonphotooxidizable P700 due to acceptor side limitation (YNA). For this discrimination, the fully oxidized P700 signal amplitude was obtained in PSII-inhibited HA/DCMU samples measured under identical conditions.

## Supplemental data

The following materials are available in the online version of this article.

**Supplemental Figure S1.** Setting for H_2_ measurement by BlueSens (GmbH, Herten, Germany).

**Supplemental Figure S2.** Exemplary measurements of total electron transfer rate (ETR) are shown.

**Supplemental Figure S3.** The electron transfer rate (ETR) was calculated via the dark pulse method after 20-min dark adaptation for strains *pgr5/lhca2*, *pgr5*, *lhca2*, and WT cc124.

**Supplemental Figure S4.** P700 deconvolution in WT cc124, single mutants *lhca2* and *pgr5*, and *pgr5/lhca2* double mutants under anoxia.

**Supplemental Figure S5.** The electron transfer rate (ETR) was calculated via the dark pulse method after 20-min dark adaptation for strains *pgr5/lhca9*, *lhca9*, and WT cc124.

**Supplemental Figure S6.** The electron transfer rate (ETR) was calculated via the dark pulse method after the 700-ms dark period for strains *pgr5/lhca9*, *lhca9*, and WT cc124.

**Supplemental Figure S7.** Schematic view on PSI remodeling processes.

## Supplementary Material

kiac055_Supplementary_Data

## Notes

F.B and M.H. designed the research; T.T.H.H., C.S., T.E., V.F, and K.Z. performed the research; T.T.H.H, T.E., K.Z., M.S., I.Y., F.B., and M.H. analyzed the data; and M.H. wrote the paper with the help of T.T.H.H. and F.B.

The author responsible for distribution of materials integral to the findings presented in this article in accordance with the policy described in the Instructions for Authors (https://academic.oup.com/plphys/pages/general-instructions) is Michael Hippler (http://mhippler@uni-muenster.de).
