# Supplementary material for: Photosystem I light-harvesting proteins regulate photosynthetic electron transfer and hydrogen production
Source: Plant Physiol. 2022 Feb 14;189(1):329–43. doi: 10.1093/plphys/kiac055 (PMC9070821; doi:10.1093/plphys/kiac055)
Supplement: kiac055_Supplementary_Data [file kiac055_supplementary_data.pdf]

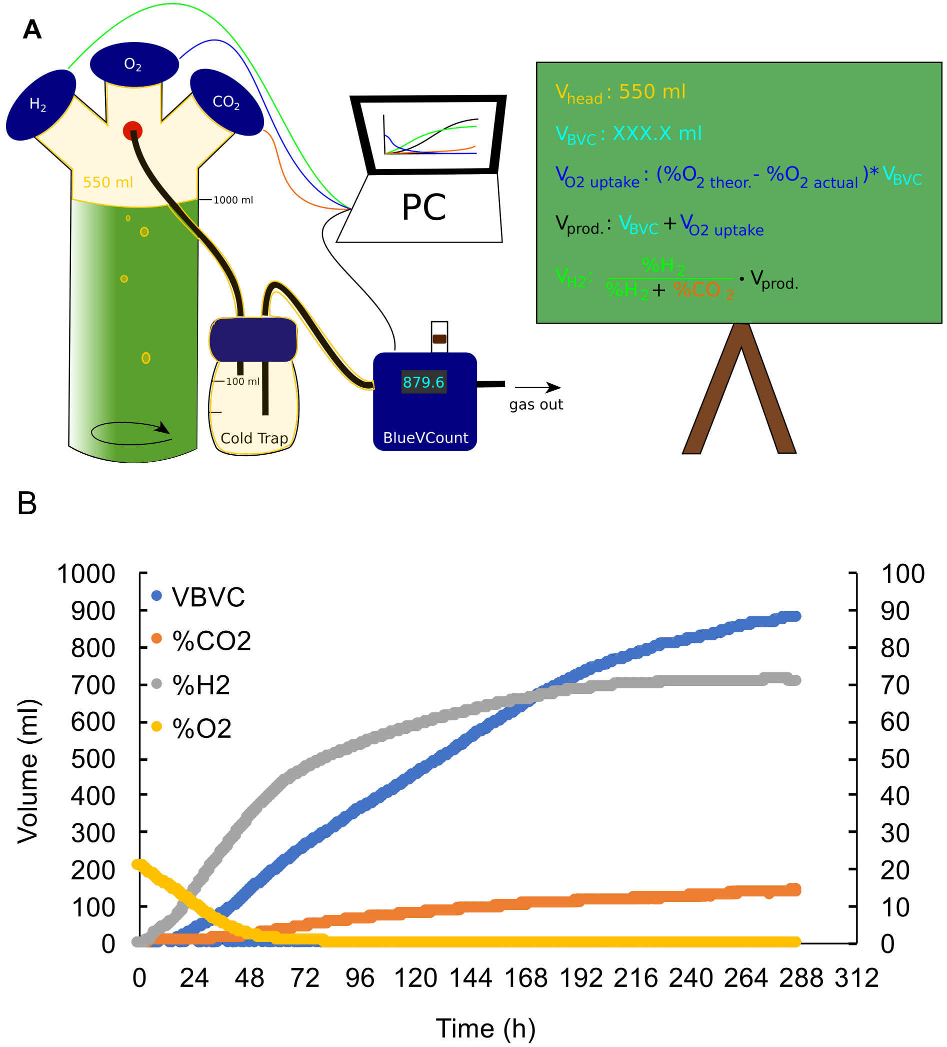

Supplemental Figure S1

Setting for H_2 measurement by BlueSens (GmbH, Herten, Germany). (A) The scheme for measuring and calculating the produced H_2 volume. H_2 production from 1 L of culture was measured under constant stirring in a gas tight glass fermenter that was equipped with three gas sensors (H_2, O_2, CO_2) constantly recording the composition of the headspace (in Vol%). Additionally, a gas volume counter (BlueVCount, BVC) was connected via a cold trap to determine the total volume of gas produced (V_{BVC}). The headspace volume (V_{head}, circled in yellow) equaled 550 ml, including the headspace in the fermenter, the cold trap bottle and tubings. For calculation of produced H_2 volume, first the volume of cellular O_2 uptake ($V_{O2\ uptake}$, blue curve) was calculated via the difference between theoretically remaining O_2 (% $O_{2\ theor}$)actually measured O_2 (% $O_{2\ actual}$) and total volume of gas produced (V_{BVC}). Second, the total gas produced (V_{prod}) was calculated from the sum of gas leaving the fermenter (V_{BVC}) and $V_{O2uptake}$. Finally, the volume of produced H_2 (V_{H2}) was calculated by relating the fraction of H_2 produced (green curve, %H_2) to CO_2 produced (orange curve, %CO_2) and and to V_{prod} for each time step. The total volume of produced H_2 was calculated by summing up the volumes of H_2. (B) Example of gas production measured by BlueSens, in which the percent of $CO_{2,}$ H_2, O_2 gases and V_{BVC} is shown by orange, grey, yellow and blue curves, respectively. The left axis shows the volume of total gas produced (V_{BVC}) while the right axis shows the percent of each gases measured in the headspace.

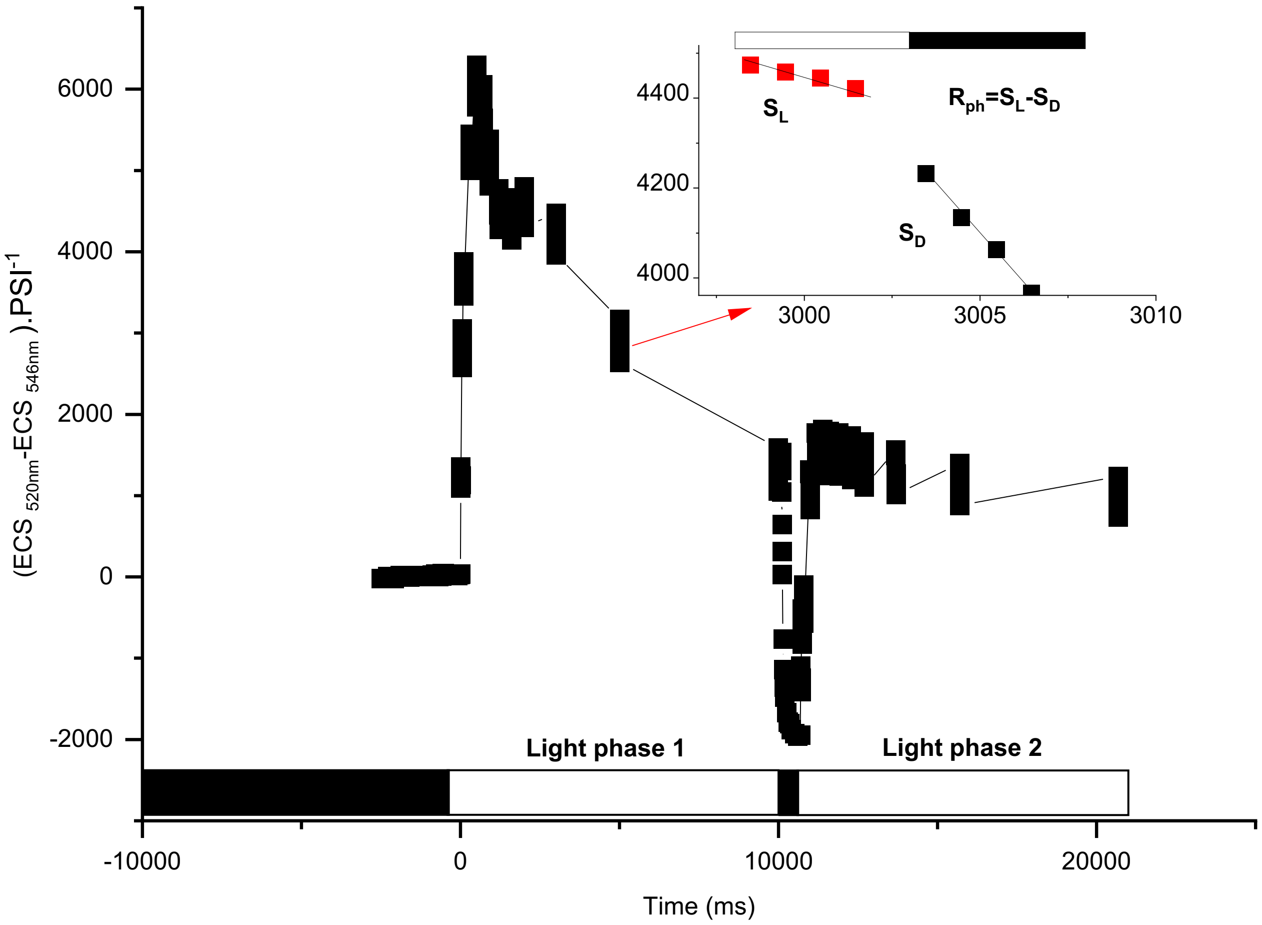

Supplemental Figure S2

Exemplary measurement of total electron transfer rate are shown. The signals of the electrochromic shift (ECS, band shift at 520 nm - 546 nm) induced by continuous illumination was measured before and during short dark periods, using the so-called dark pulse method. The first light phase of 10 s is followed by 700 ms darkness and a second light phase of 10 s illumination. At each time point, the PSI photochemical rate R_{ph} (unit charge separations.PSI^{-1}.s^{-1}) were calculated by the slope of the ECS measured before (S_L) and after (S_D) the light switched off. The bars show dark (black) and light (white) phases.

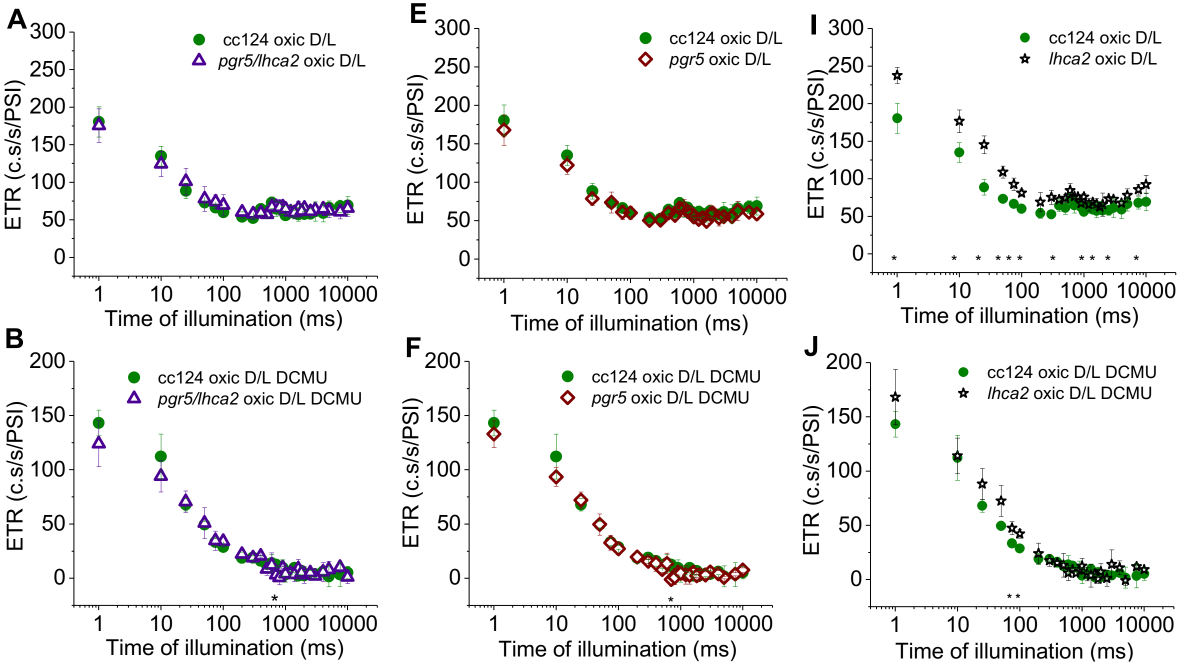

Supplemental Figure S3

The electron transfer rate (ETR) was calculated via the dark pulse method after 20 min dark adaptation for strains *pgr5/lhca2*, *pgr5*, *lhca2* and WT cc124. For reference of the measurement routine, see Supplemental Figure S2. (A), (E), (I), ETR in oxic condition (B), (F), (J), ETR in oxic conditions and in the presence of DCMU. (C), (G), (K) ETR in anoxic conditions, (D), (H), (L) ETR in anoxic conditions and in the presence of DCMU. Each time point is an average of at least three biological replicates (± SD) with statistical comparisons analyzed by Student's t-test (* $p \leq 0.05$).

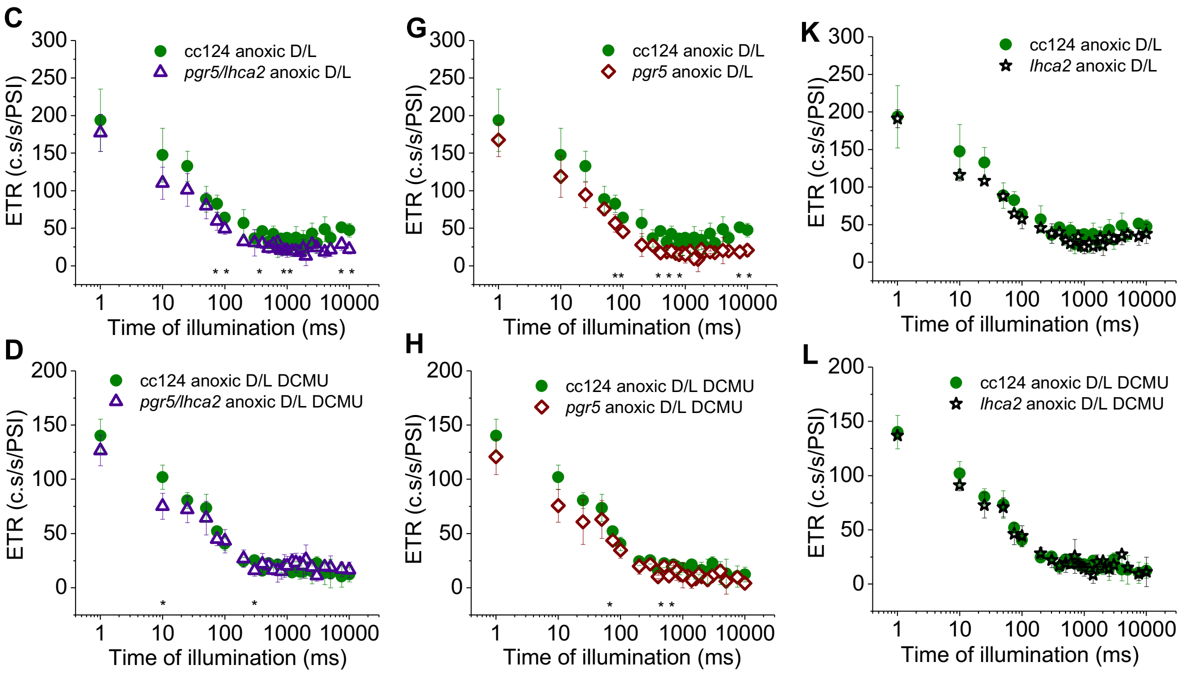

Supplemental Figure S3 (continued)

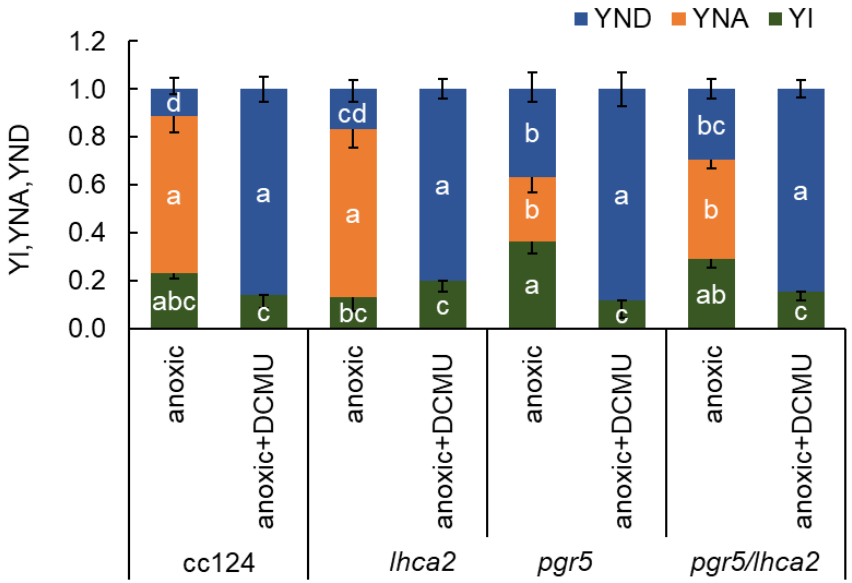

Supplemental Figure S4

P700 deconvolution in WT cc124, single mutants *lhca2, pgr5* and *pgr5/lhca2* double mutants under anoxia. The fractions of PSI as Φ PSI (YI), YNA, and YND were described for three biological replicates in the presence and absence of DCMU. The parameters were grouped using one way ANOVA as the Tukey hot doc test ($p<0.05$), with different letters indicating the significance of the difference in means (±SD).

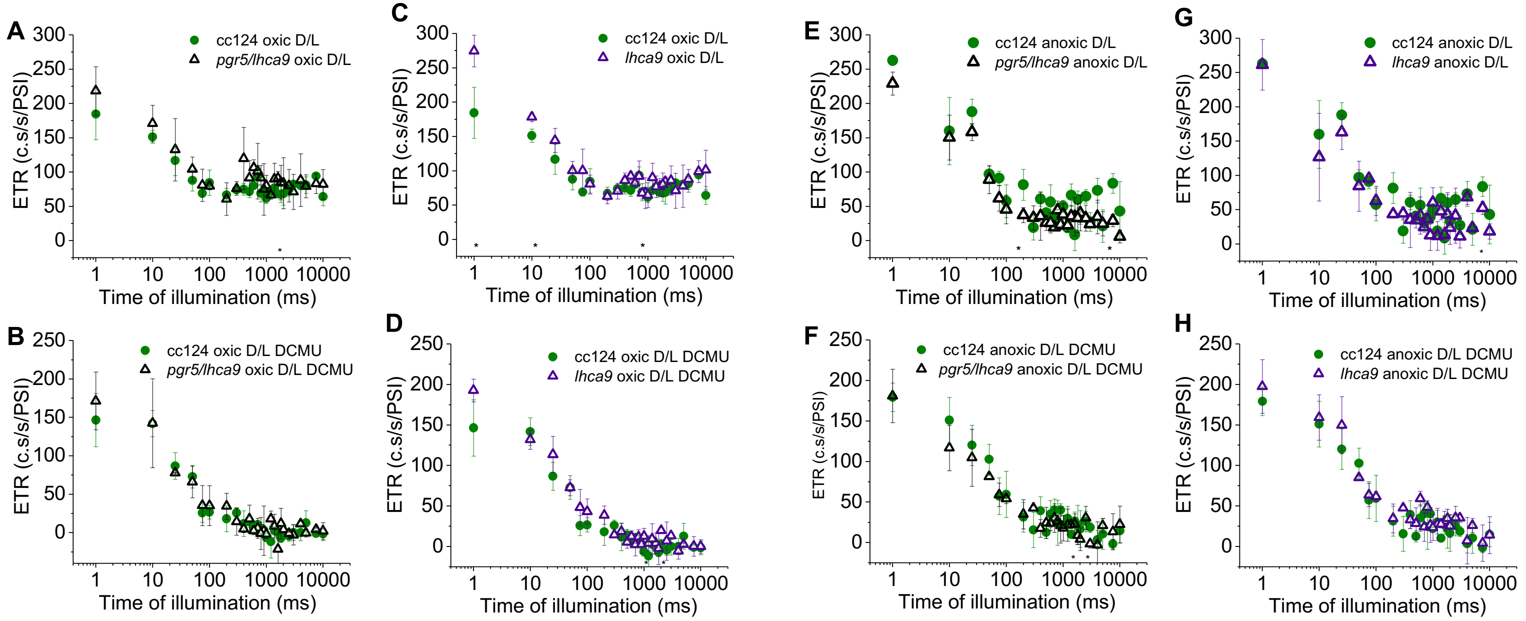

Supplemental Figure S5

The electron transfer rate (ETR) was calculated via the dark pulse method after 20 min dark adaptation for strains *pgr5/lhca9, lhca9* and WT cc124. For reference of the measurement routine, see Supplemental Figure S2. (A),(C) and (B), (D) are ETR in oxic condition under the absence and presence of DCMU, respectively. (E), (G) and (F), (H) are ETR in anoxic condition under the absence and presence of DCMU, respectively. Each time point is an average of three biological replicates (± SD) with statistical comparisons analyzed by Student's t-test (* $p \leq 0.05$).

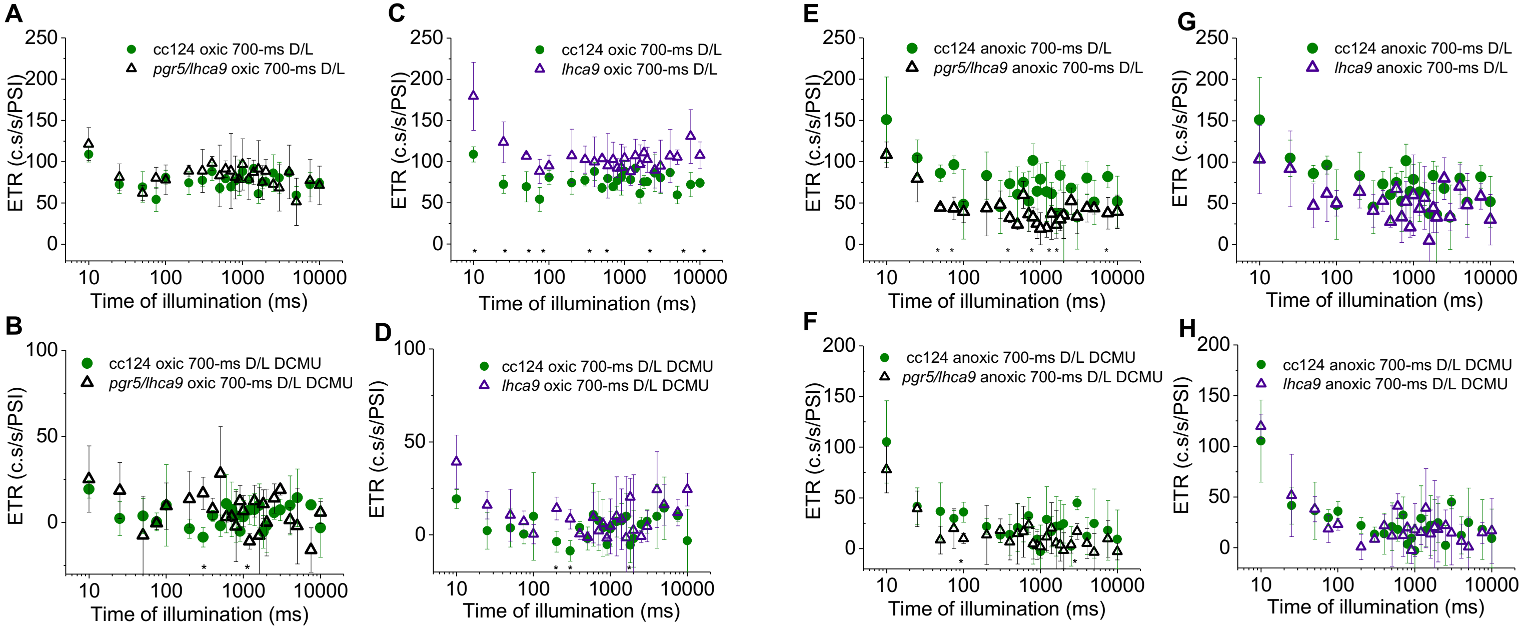

Supplementary Figure S6

The electron transfer rate (ETR) was calculated via the dark pulse method after the 700 ms dark period for strains *pgr5/lhca9, lhca9* and WT cc124. For reference of the measurement routine, see Supplemental Figure S2. (A),(C) and (B), (D) are ETR in oxic condition under the absence and presence of DCMU, respectively. (E), (G) and (F), (H) are ETR in anoxic condition under the absence and presence of DCMU, respectively. Each time point is an average of three biological replicates (± SD) with statistical comparisons analyzed by Student's t-test (* $p \leq 0.05$).

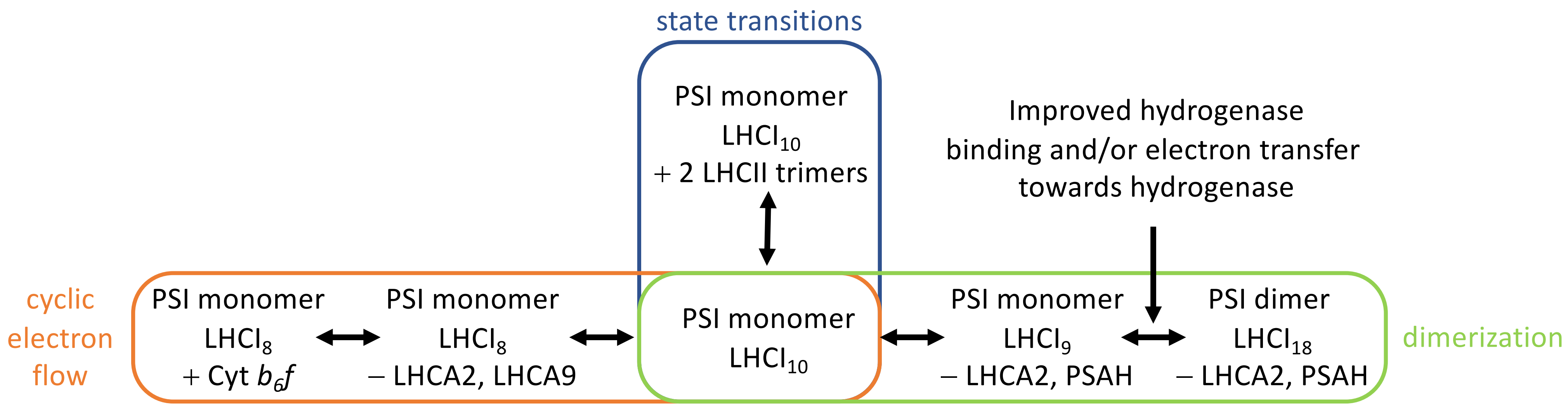

Supplemental Figure S7

Schematic view on PSI remodeling processes. We suggest that the absence of LHCA2 enhances H_2 photo-production via improved binding of hydrogenase to PSI and/or electron transfer towards hydrogenase via FDX1 after its photoreduction by PSI.
